# Functional analysis of *PHYB* polymorphisms in *Arabidopsis thaliana* collected in Patagonia

**DOI:** 10.3389/fpls.2022.952214

**Published:** 2022-09-07

**Authors:** María Jimena Ruiz-Diaz, Daniel Matsusaka, Jimena Cascales, Diego H. Sánchez, Maximiliano Sánchez-Lamas, Pablo D. Cerdán, Javier F. Botto

**Affiliations:** ^1^IFEVA (CONICET-UBA), Facultad de Agronomía, Universidad de Buenos Aires, Buenos Aires, Argentina; ^2^Fundación Instituto Leloir, IIBBA-CONICET, Buenos Aires, Argentina

**Keywords:** natural genetic variation, Arabidopsis, QTL mapping, shade avoidance response, *PHYB*

## Abstract

*Arabidopsis thaliana* shows a wide range of natural genetic variation in light responses. Shade avoidance syndrome is a strategy of major adaptive significance that includes seed germination, elongation of vegetative structures, leaf hyponasty, and acceleration of flowering. Previously, we found that the southernmost Arabidopsis accession, collected in the south of Patagonia (Pat), is hyposensitive to light and displays a reduced response to shade light. This work aimed to explore the genetic basis of the shade avoidance response (SAR) for hypocotyl growth by QTL mapping in a recently developed 162 RIL population between Col-0 and Pat. We mapped four QTL for seedling hypocotyl growth: *WL1* and *WL2* QTL in white light, *SHADE1* QTL in shade light, and *SAR1* QTL for the SAR. *PHYB* is the strongest candidate gene for *SAR1* QTL. Here we studied the function of two polymorphic indels in the promoter region, a GGGR deletion, and three non-synonymous polymorphisms on the *PHYB* coding region compared with the Col-0 reference genome. To decipher the contribution and relevance of each *PHYB*-Pat polymorphism, we constructed transgenic lines with single or double polymorphisms by using Col-0 as a reference genome. We found that single polymorphisms in the coding region of *PHYB* have discrete functions in seed germination, seedling development, and shade avoidance response. These results suggest distinct functions for each *PHYB* polymorphism to the adjustment of plant development to variable light conditions.

## Introduction

Light responses of plant populations differ between contrasting habitats and show adaptive variation (Maloof et al., 2001; Alonso-Blanco et al., 2009). Different physiological responses are mediated by light perception through photoreceptors including phytochromes, cryptochromes, phototropins, and UVR8 (Chen et al., 2004; Bae and Choi, 2008; Legris et al., 2019). In Arabidopsis, the phytochromes are encoded by *PHYA, PHYB, PHYC, PHYD,* and *PHYE* genes. Phytochromes (phys) perceive red (R) and Far-red (FR) signals and induce seed germination, seedling de-etiolation, and shade avoidance responses, among others. Light can promote seed germination through a Red/Far-Red (R/FR) reversible response principally by phyB and secondarily by phyC, phyD, and phyE (Bae and Choi, 2008; Tognacca and Botto, 2021). After germination, the light signal transduced by phytochromes and cryptochromes triggers seedling de-etiolation, inhibits hypocotyl growth, and promotes the opening and greening of cotyledons (Bae and Choi, 2008; Legris et al., 2019). Then, young plants compete for photosynthesis light with neighbors. The plant canopy efficiently absorbs blue and R photons by photosynthetic pigments and increases the relative amount of FR photons. Plants perceive R/FR ratio changes even before being shaded by other plants, enhancing the elongation of vegetative structures, leaf hyponasty, and acceleration of flowering, collectively known as shade avoidance responses (SARs; Casal, 2013; Ballaré and Pierik, 2017).

A huge natural genetic variation in SARs has been pioneeringly documented in Arabidopsis (Botto and Smith, 2002). A higher light sensitivity to shade was documented for flowering and dry biomass in coastal than mountain populations of Arabidopsis being the SAR plasticity increased with higher temperatures and lower rainfall occurring on the coast (Botto, 2015). Latitudinal origin of natural populations of Cardamine is also responsible for differences in light sensitivities being higher latitude species more sensitive to red light than lower latitude sister (Ikeda et al., 2021). The genetic architecture of the SARs has been deciphered through quantitative genetics using mapping populations such as recombinant inbred lines (RILs), nested association mapping (NAM) population (Ta et al., 2020), or genome-wide association study (Filiault and Maloof, 2012). By Quantitative Trait Loci (QTL) mapped in different RIL populations, several studies have proposed candidate genes for SARs (Jiménez-Gómez et al., 2010; Coluccio et al., 2011; Kasulin et al., 2013; Jiang et al., 2019; Ta et al., 2020). For example, *PHYB* has been associated as a gene candidate to detect low plant density in the Ler × No-0 and Ler × Cvi RIL populations (Botto and Coluccio, 2007), and *ELF3* was documented as the SAR causal gene for hypocotyl growth and flowering in the Bay × Sha RIL population (Jiménez-Gómez et al., 2010; Coluccio et al., 2011). Interestingly, two close mapped genes, *PHYB* and *ERECTA,* have been suggested as candidate genes for the SAR regarding the genetic background in three RIL populations sharing Col-0 as the same parental line (Kasulin et al., 2013). Together these results suggest that the SAR includes complex traits associated with the genetic background in combination with the environmental circumstances experienced by plants. Similar conclusions arrived after studying the genetic architecture of SARs in late-vegetative and reproductive traits using a NAM population of Arabidopsis plants (Ta et al., 2020).

The principal R-light photoreceptor mediating the SARs is the phyB being its holoprotein relatively stable (Legris et al., 2019). PhyB is synthesized in the Pr inactive form and photo-transformed to the Pfr active form upon absorption of R photons. Plant phytochromes are dimeric, each monomer consists of ~1,150 amino acids covalently bound to its chromophore, a linear tetrapyrrole named phytochromobilin (PΦB). The PHYB apoprotein has an N-terminal photosensory module (PSM), which consists of the N-terminal extension (NTE), and three structurally related domains known as PAS (Period/Arnt/SIM), GAF (cGMP phosphodiesterase/adenylyl cyclase/FhlA) and PHY phytochrome-specific domain. The C-terminal module (CTM) contains two PAS domains and a histidine kinase-related domain (HKRD). The chromophore is bound covalently to a conserved cysteine in the GAF domain, which has intrinsic chromophore lyase activity (Burgie et al., 2014). After light perception, the isomerization and conformational change of the PΦB chromophore leads to a cascade of structural modifications in the protein by the interaction with the GAF domain by non-covalent interactions with residues from the rest of PSM domains (Burgie et al., 2014). The Initial conformational rearrangements in the PSM lead to structural changes affecting the CTM. Ultimately, the light-induced Pfr interacts with other proteins necessary for nuclear localization and signaling, such as PHYTOCHROME INTERACTING FACTOR (PIF) transcription factors (Leivar et al., 2012), ubiquitin E3 ligase complexes (Podolec and Ulm, 2018), and SUMOylation complexes (Sadanandom et al., 2015).

A natural variation study of the *PHYB* gene in 33 accessions of Arabidopsis found 14 non-synonymous polymorphisms mapped in the coding region (Filiault et al., 2008). Four polymorphisms were mapped on the PSM module while 10 polymorphisms were located on the CTM module (Filiault et al., 2008). Seven of those non-synonymous polymorphisms were documented between Ler and Cvi accessions. By transgenic analysis, the complete Cvi-*PHYB* variant in the *phyB-9* mutant background caused a low red-light sensitivity in seedling de-etiolation compared with the Ler-*PHYB* allele (Filiault et al., 2008). Recently, it has been demonstrated that fine tuning Pfr stability is a fundamental mechanism for plants to optimise phytochrome-related traits in a comparative study between two sister species of Cardamine, a close Brassicaceae relative of Arabidopsis. In fact, the higher latitude species (*Cardamine bellidifolia*; *Cb*) is more sensitive to R light compared with its lower latitude sister (*C. nipponica*; *Cn*). Moreover, *CbPHYB* is less sensitive to high temperatures in the *phyB*-deficient mutant of *Arabidopsis thaliana* than *CnPHYB*: that is Pfr CbphyB was more stable in nuclei than CnphyB (Ikeda et al., 2021). However, we lack information about the individual contribution of each *PHYB* polymorphism in the diversity of plant light responses.

The native range of Arabidopsis distribution is Eurasia and North Africa (Al-Shehbaz and O'Kane, 2002; Durvasula et al., 2017). Arabidopsis has been introduced worldwide, especially around the Northern Hemisphere (Exposito-Alonso et al., 2019). In South America, Arabidopsis was probably introduced in the last century together with the first European immigrants arriving in the Occidental District of Patagonia. Patagonia germplasm (Pat) represents a single haplogroup (Kasulin et al., 2017). In the field, Pat showed a strong plasticity according with the place of collection. Accelerated flowering plants with low dry biomass were collected behind shrubs, while still green plants with profuse lateral branching were found in open areas (Kasulin et al., 2017). In the laboratory, Pat plants show low sensitivity to light and have a strong vernalization requirement for flowering that correlates with impaired expression of phytochrome and FLC signaling-related genes (Kasulin et al., 2017). Here, we hypothesize that the contrasting light responses between Col-0 and Pat could be explored to study the genetic architecture of the SAR. We mapped QTLs in the SAR mediating hypocotyl growth by using a new genetic resource of the Col-0 × Pat RIL population (Matsusaka et al., 2021). We found *SAR1* QTL mapped on chromosome II, being *PHYB* the strongest candidate gene. Then, we examined the contribution of individual or combined Pat-*PHYB* polymorphisms on physiological responses mediated by the phyB using Col-0 as the reference genome. We demonstrated that individual polymorphisms on the coding region of *PHYB*, but not in the promoter, have discrete functions in seed germination, seedling de-etiolation, and shade avoidance.

## Materials and methods

### Plant material and growth conditions

Arabidopsis plants were grown under long-day conditions (16 h light/8 h dark, PAR = 100 μmol m^−2^ s^−1^) with an average temperature of 21 ± 2°C. Seeds of each genotype were harvested as a single bulk consisting of at least 5 plants. Seeds were stored in open tubes inside a closed box that was maintained in darkness with silica gel at 4°C until experiments were performed. We used Columbia-0 (Col-0), Pat, and other accessions sharing the same Pat-*PHYB* polymorphisms (Supplementary Table S1). Heterogenous inbred families (HIF20 and HIF71) were used to confirm *SHADE1* QTL and developed previously (Matsusaka et al., 2021). The seeds used in this work were provided by the Nottingham Arabidopsis Stock Centre (NASC).

### Physiological experiments

For the seed germination experiment, samples of 20 seeds per genotype were sown in clear plastic boxes (40–33-mm^2^ 15-mm height), each containing 3 ml of 0.8% (w/v) agar in demineralized water. The seeds were incubated at 7°C in darkness for 3 days. Then, the seeds were treated with 30 min pulse of either FR (42 μmol m^−2^ s^−1^) or R (30 μmol m^−2^ s^−1^, Supplementary Figure S1). The FR pulse established a photoequilibrium (Pfr/Pr + Pfr) of 0.03 and the R pulse of 0.87 in the seed tissues (Botto et al., 1995, 1996). Then, the seeds were incubated in darkness at 25°C until germination was counted after 4 days. The criterion for germination was the emergence of the radicle, and the average germination of 20 seeds in each box was a replicate. The R/FR response (%) was calculated as the difference between germination in R pulse minus FR pulse.

For the seedling de-etiolation experiment, samples of 15 seeds per genotype were sown in transparent plastic boxes and stratified as indicated above. Then, the seeds were exposed to a 2 h-R pulse and kept in darkness for the promotion of seed germination. After 24 h in darkness, the seedlings were exposed to continuous R (Rc = 30 μmol m^−2^ s^−1^, Supplementary Figure S1) or kept in darkness at 22°C. On the fourth day, the hypocotyl length was measured for the 10 tallest seedlings in each box (a replicate). We estimated the hypocotyl length in red continuous relative to darkness to have a real measure of the seedling de-etiolation response for each genotype. For other references see Matsusaka et al. (2021).

For the SAR experiment, samples of 15 seeds per genotype were sown in transparent plastic boxes and stratified as indicated above. Then, the seeds were exposed to a 2 h-R pulse and kept in darkness for the promotion of seed germination. After 24 h of darkness, the seedlings were transferred to continuous white light at 22°C for complete seedling de-etiolation. After 2 days, seedlings were transferred to white light with incandescent lamps (WL) or simulated shade with a photoperiod of 10 h light/14 h darkness for 5 days. In WL, the PAR (Photosynthetic Active Radiation) was 130 μmol m^-2^ s^-1^ (*R* = 13, FR = 12, and *B* = 9 μmol m^−2^ s^−1^, Supplementary Figure S1). The simulated shade was obtained with a green acetate filter (code #089, Lee Filters, http://www.leefilters.com) that established the PAR = 14 μmol m^−2^ s^−1^ (*R* = 0.27, FR = 2.72, and *B* = 1.20 μmol m^−2^ s^−1^, Supplementary Figure S1). The R/FR ratio was 1.1 in WL and 0.10 in the simulated shade condition. On the fifth day, the hypocotyl length was measured for the 10 tallest seedlings in each box (a replicate). We estimated the SAR index as the ratio between the hypocotyl length in simulated shade and WL.

At the end of the de-etiolation and SAR experiments, we photographed the seedlings with a digital camera (PowerShot; Canon, Tokyo, Japan), and the hypocotyl length was determined using the Image J program (Schneider et al., 2012). Light measurements were done with a Spectroradiometer SPECTROSENSE2/2 + Meter (Skye Instruments Ltd., Powys, United Kingdom). In all cases, data were statistically analyzed by ANOVA and paired comparisons by Bonferroni’s test. For the analysis we used the Info Stat Software version 2017 (Grupo Info Stat, FCA, Universidad Nacional de Córdoba, Argentina).

### QTL mapping in shade avoidance

For QTL mapping in SAR, the hypocotyl length values of seedlings exposed to WL and simulated shade were used as the average of three replicates for each RIL and 12 replicates for parental lines. Each replicate was the average of the 10 tallest seedlings. To perform QTL mapping, we initially applied Box-Cox of MASS library transformation to turn phenotypic non-normal residuals into a normal distribution with homoscedasticity. With lme4 package, we extracted the fitted values from the best model calculating the best-linear-unbiased estimates (BLUEs) and best-linear-unbiased-predictions (BLUPs). These were used for quantitative-trait-loci (QTL) mapping, conducted with the R/qtl package (Arends et al., 2010). We removed non-informative markers with drop.nullmarkers function, used est.rf function to estimate pairwise recombination fractions between all marker pairs and plot them, and applied calc.genoprob function to calculate conditional genotypes probabilities given the multipoint marker data with possible allowance for genotyping errors. Subsequently, composite-interval-mapping (CIM) with “hk” method was applied to calculate the genetic interval coordinates and the LOD (Logarithm-of-the-Odds score). LOD was considered significant when *α* < 0.1 after 10,000 permutations performed with n.perm function (Arends et al., 2010). For other references see Matsusaka et al. (2021).

### Exploration of candidate genes

We explored candidate genes for those mapped into the QTLs. Having no *a priori* evidence that variation is based on differential expression or structure, we focused on analyzing each gene’s Pat SNPs, clustering polymorphisms according to SnpEff high functional impact categories such as missense variant, splice donor variant, splice acceptor variant, start lost, stop gained and stop lost codon (Cingolani et al., 2012). We then searched and retrieved light-regulated relevant genes within QTLs from manually-curated-published reports and GO terms analysis using different databases annotated coding features. For additional references see Matsusaka et al. (2021).

### Construction and selection of *PHYB* transgenic plants

The genomic sequence of *PHYB* locus was extracted from *Arabidopsis thaliana* chromosome II sequence (Genbank Accession number NC 003071) and the sequence of phyB cDNA was obtained from Genbank Accession number NM_127435.3. The *PHYB* transgenic lines were generated as previously described Sánchez-Lamas et al. (2016). Briefly, the phytochrome cDNAs were obtained by retrotranscription from Col-0 and Pat RNA and cloned into the pCHF5 plasmid fused to the C-terminus HA tag. Single locus insertion lines from the T3 generation were selected. Two independent transgenic events for each type of polymorphism were generated. For cloning, the complete coding sequence of *PHYB* was PCR-amplified from retrotranscribed cDNA obtained by retrotranscription of total RNA. Five prime and 3′ primers included BamHI and XbaI sites to clone the fragments in pBluescript vector. Before cloning, relevant restriction sites were eliminated from the coding *PHYB* region by introducing silent mutations by using PCR with specific primers. We used a similar approach to introduce the Pat polymorphisms in the Col *PHYB* sequence. Fragments were then assembled by fusion-PCR, and restriction sites were added to both 5′ and 3′ ends with primers bearing BamHI and XbaI sites as described above. Each cDNA was cloned in the pBlueScript plasmid as a BamHI-XbaI fragment, and the sequence was confirmed by Sanger sequencing. Finally, each fragment was subcloned in the CHF5 binary vector as a BamHI-SalI fragment, under the CAMV 35S promoter and with a HA tag fused to the C-terminus. Primers used in this study are listed in Supplementary Table S2.

### *PHYB* gene expression analysis

Samples were harvested in liquid nitrogen 3 h after the beginning of day 4. Total RNA was extracted with the Spectrum Plant Total RNA Kit (Sigma-Aldrich) and subjected to a DNAse treatment with RQ1 RNase-Free DNase (Promega). cDNA derived from this RNA was synthesized using Invitrogen SuperScript III and an oligo-dT primer. The synthesized cDNAs were amplified with FastStart Universal SYBRGreen Master (Roche) using the 7500 Real-Time PCR System (Applied Biosystems, available from Invitrogen). The UBIQUITIN-CONJUGATING ENZYME 10 (UBC10) gene was used as the normalization control.

### LUC bioluminescence assay

For the LUC bioluminescence assay, Col-0 and Pat were transformed with the p.PHYBpat::LUC and p.PHYBCol::LUC reporter using the floral dip method. Seedlings were grown directly on half-strength Murashige and Skoog 0.8% agar medium supplemented with 1% sucrose in a 96-well plate. One seed was placed per well and the seedlings were entrained under 16-h light/8-h dark cycles. After 7 days, 40 μl of luciferin (20 mM) was added to each well. The plate was transferred to constant light conditions and dark conditions and placed in a microplate luminometer LB-960 (Berthold Technologies, Bad Wildbad, Germany) to measure the bioluminescence emitted by each seedling every hour. Data analysis was conducted using the Mikrowin 2000 software (version 4.29, Labsis Laborsysteme GmbH, Neunkirchen-Seelscheid, Germany). Period estimates were calculated with Brass 3.0 software and analyzed using FFT-NLLS.

### Protein extraction and blots

Arabidopsis seedlings were ground in liquid nitrogen and resuspended in cold extraction buffer (50 mM Tris–HCl pH 7.5, 150 mM NaCl, 0.1% p/v NP40, 10% glycerol) at a ratio of 1 g of tissue per ml of extraction buffer. The extract was centrifuged at 13,000 *g* for 30 min. When necessary, aliquots were stored at −80°C to quantify proteins by the method of Lowry. When used for gel loading, one volume of 2X SDS sample buffer was added to each extract and boiled for 5 min.

For immunoblot, proteins were run on 8%–10% SDS-PAGE and transferred to a nitrocellulose membrane (Hybond-ECL, Amersham Biosciences, RPN303D). The HA tag was detected with a monoclonal anti-HA peroxidase (Roche 3F10, 2013819) at a dilution of 1:500. The signal was developed with the Millipore chemiluminescent HRP substrate kit.

For dotblots, the indicated extracts and dilutions were spotted onto nitrocellulose membrane (Hybond-ECL, Amersham Biosciences, RPN303D) and developed with the monoclonal anti-HA peroxidase (Roche 3F10, 2013819), as described above. Luminiscence was imaged with an ImageQuant™ LAS 4000 (GE Healthcare).

## Results

### Col-0 and Pat phenotypes in shade avoidance

**Figure 1:**
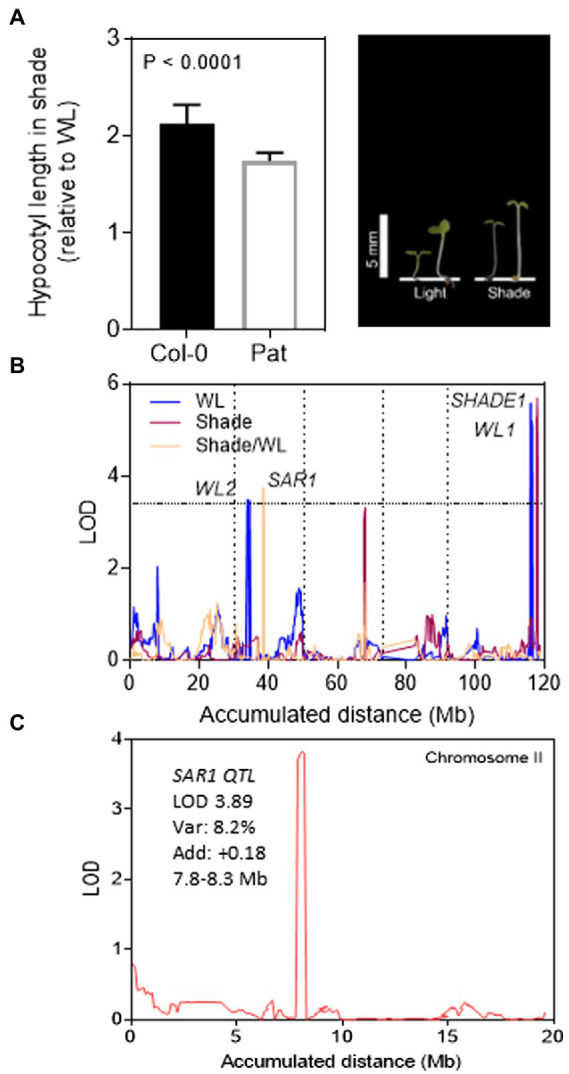
QTL mapping for shade avoidance response in Col-0 and Pat RIL population. **(A)** SAR index for Col-0 and Pat seedlings calculated as the ratio of hypocotyl length in simulated shade and WL. The photo shows representative Col-0 (left) and Pat (right) seedlings in WL and simulated shade. **(B)** QTL mapping for hypocotyl growth in WL, simulated shade, and SAR index in the Col-0 and Pat RIL population. LOD score is shown for the accumulated distance of the five chromosomes separated by vertical dashed lines. The horizontal dashed line indicates the LOD significance threshold value. **(C)** LOD score is shown for the absolute distance of chromosome II (Mb). *PHYB is the candidate gene* mapped into the confidence interval of *SAR1* QTL. For *SAR1* QTL, is indicated the LOD, % explained variability, additive effects (positive additive effects mean a higher contribution of Col-0 alleles) and confidence interval.

We studied the hypocotyl growth of Col-0 and Pat seedlings in the SAR. After de-etiolation, we transferred the transparent boxes with seedlings to WL or simulated shade for additional 5 days. In both light conditions, the hypocotyl length was shorter in Col-0 than Pat being the effects higher in WL (Figure 1A). We calculated the SAR index as the ratio between the hypocotyl length in simulated shade and WL. As expected, the SAR index was higher in Col-0 than in Pat (*p* < 0.0001; 2.11 ± 0.02 and 1.74 ± 0.06, respectively). These results confirm that Pat seedlings are less responsive to light than Col-0 (Kasulin et al., 2017; Matsusaka et al., 2021).

### QTL mapping for shade avoidance

Regarding the shade avoidance contrasting phenotypes between Col-0 and Pat, we mapped QTLs for hypocotyl length in WL, simulated shade, and SAR index in a new re-sequenced RIL population of Col-0 × Pat (Matsusaka et al., 2021). A wide phenotypic transgression was found for the three traits in the RIL population being wider in WL than simulated shade and SAR index (Supplementary Figure S2). The broad-sense heritability ranged between 0.50 and 0.83 confirming that enough genetic variation can be explored by QTL mapping (Supplementary Table S3). We mapped 2 QTLs in WL (*WL1* and *WL2*), one QTL in the simulated shade (*SHADE1*), and another QTL for SAR index (*SAR1*; Figure 1B; Table 1). *WL1* and *SHADE1* QTL mapped close together on the lower arm of chromosome V, and *WL2* and *SAR1* QTL mapped on chromosome II. The phenotypic variation explained for each QTL ranged between 15 and 7% (Table 1). *SHADE1* QTL (LOD = 6.3, Var = 14.6%) was also mapped for hypocotyl length in seedling de-etiolation in darkness, blue and FR continuous light (Matsusaka et al., 2021).

**Table 1:** QTL mapping for WL, simulated shade, and SAR index calculated as the ratio of hypocotyl length in simulated shade and WL in the Col-0 and Pat RIL population.

Chr	QTL	Trait	Map Pos (cM)	Interval (cM)	Interval (kb)	LOD	Var (%)	Add
5	*SHADE1*	Shade	82.4	81.2–83.7	25.700–26.200	6.3	14.6	−0.56
5	*WL1*	WL	78.1	77.3–78.3	23.700–24.400	5.9	11.5	−0.35
2	*SAR1*	Shade/WL	29.2	28.8–30.5	7.800–8.300	3.8	8.5	0.18
2	*WL2*	WL	17.6	17.4–18.1	3.500–5.300	3.5	7.2	−0.30

The table indicates the significance of the QTL (*α* = 0.10), the chromosome number, trait, map position (cM), 2-LOD interval in physical distance (bp), LOD, % variability explained, and additive effects (the positive and negative signs indicate Col-0 alleles and Pat alleles increase the average response of the trait, respectively). The number of QTL is arbitrary following the relevance of the LOD value.

### Exploration of candidate genes for shade avoidance

The re-sequencing of the new RIL population enabled the unambiguous physical anchoring of ultra-high-density molecular markers, resulting in shorter genetically associated intervals bearing in principle a reduced set of potentially causative genes. For each QTL, we found between 132 and 399 annotated Col-0 genes (Supplementary Table S4). Subsequently, variant-call scrutiny of deep sequenced Pat genome compared to Col-0 allowed the recognition of potentially functional significant Pat alleles. We documented 323 transcripts (260 annotated coding features), in the four QTLs, presented high impactful Pat SNPs such as missense, nonsense, splice donor or splice acceptor variants, and start and stop lost codons (Supplementary Table S5). We searched and retrieved light-regulated relevant genes within QTLs from manually curated published reports and GO terms analysis. Cross-examination of this information finally revealed three annotated coding features in *SAR1* QTL and one gene in *SHADE1* QTL (Supplementary Table S6) exhibited documented roles in light responses and at the same time impactful Pat alleles.

We confirmed the additive effects of *SHADE1* QTL by two independent HIFs (Heterogeneous inbred families, HIF20 and HIF71, Supplementary Figure S3). According to the QTL mapping output, the Pat alleles increased the hypocotyl length in HIF20 and HIF71 seedlings cultivated under simulated shade. *PHYB* maps within the confidence interval of *SAR1* QTL (LOD = 3.9, Var = 8.2%) and, as expected, the Col-0 alleles increase the SAR (Figure 1C; Table 1). Interestingly, *SAR1* QTL mapped close to *RED/DARK* QTL found previously for seedling de-etiolation in red light (Matsusaka et al., 2021). Using the ultra-high-density SNPs markers in this region, we found that *SAR1* QTL did not colocalize with *RED/DARK* QTL for seedling de-etiolation in the R/dark trait (Supplementary Figure S4).

### Natural variation for *PHYB* polymorphisms between Col-0 and Pat

**Figure 2:**
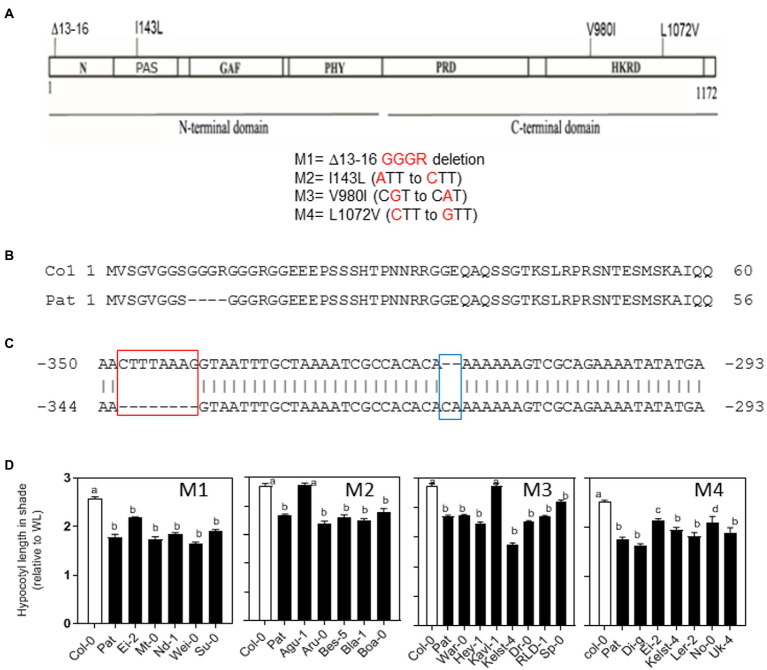
*PHYB* polymorphisms between Col-0 and Pat accessions. **(A)** *PHYB* polymorphisms in the coding region between Col-0 and Pat. The diagram shows the four polymorphisms in the N-and C-termini domains (M1 = deletion, M2 = I143L, M3 = V980I, and M4 = L1072V). **(B)** Col-0 and Pat amino-acid sequences show the GGGR deletion of Pat in the amino termini of PHYB protein. **(C)** The two indels polymorphisms in the promoter of *PHYB* gene between Col-0 and Pat tested in this study. The deletion of 8 nucleotides at position −341/−334, and the insertion of 2 nucleotides at position −318/−319 in the Pat promoter up to ATG starting codon. **(D)** Natural variation for hypocotyl SAR index calculated as the ratio of hypocotyl length in simulated shade and WL between accessions sharing the same *PHYB-*Pat polymorphism at the positions M1, M2, M3, and M4. Significant differences between means are shown with different letters by one-way ANOVA followed by Bonferroni post-test (*p* < 0.05). Each bar represents mean ± SE (*n* = 6).

We hypothesized that *PHYB* is the strongest gene candidate for *SAR1* QTL because (i) *PHYB* gene mapped into the confidence interval of *SAR1* QTL interval containing 132 genes (Supplementary Table S4), (ii) *PHYB* gene has been documented to have a prominent function in SAR, and (iii) Pat seedlings show reduced response to light and partially resemble the *phyB*-mutant phenotype for hypocotyl length in WL. Firstly, we found SNPs in the Pat-*PHYB* into the promoter (−2,000 bp) and encoded gene comparing with Col-0 reference genome using the DNAseq of five Pat previously published genomes (one ancestral and 4 different lines collected in Patagonia, for other references see Kasulin et al., 2017). In this analysis, we found 6 variants in the promoter, 2 variants in the 5’UTR, 4 synonymous, and 3 non-synonymous polymorphisms in the cDNA including 3 variants in the introns of the Pat-*PHYB* (Supplementary Table S7). Then, we sequenced the promoter and the coding region of *PHYB*-Pat by sanger and confirmed the three non-synonymous polymorphisms in the coding region (M2, M3, and M4, Figure 2A). In addition, we identified a GGGR deletion between 13–16 amino-acid positions on the amino terminus (M1, Figures 2A,B); and a two-base insertion and eight- base deletion on the promoter of the Pat-*PHYB* (−318/−319 and −334/−341, respectively; Figure 2C). These three indels were validated by Sanger sequencing using specific primers and confirmed with available Pat DNAseq data (Kasulin et al., 2017).

We hypothesized that the four Pat-*PHYB* polymorphisms in the coding region can be conserved in other Arabidopsis accessions if they are functional in SAR. We studied the SAR for hypocotyl length in 22 accessions that share the same Pat-*PHYB* polymorphisms at the GGGR deletion between 13 and 16 amino-acids, and the three non-synonym substitutions at I143L, V980I, and L1072V positions. We selected between 5 and 7 accessions sharing a unique and common polymorphism with Pat in each of the four positions: the deletion and the I143L substitution were mapped on the N-terminal PSM, and the V980I and L1072V substitutions were mapped on the HKRD domain of the C-terminal CTM (Figures 2A,B). Globally, the seedlings from different accessions sharing the Pat polymorphisms on the *PHYB* coding region showed a reduced SAR as Pat (Figure 2D). This is true for all the accessions with the GGGR deletion and L1072V (M1 and M4, respectively), and almost all accessions with I143L and V980I polymorphisms, respectively (except for Agu-1 for M2, and Kavl-1 for M3, Figure 2D). These results suggest that the four *PHYB-*Pat polymorphisms contribute independently to the SAR of hypocotyl length in different genetic backgrounds.

### Discrete functions of *PHYB* polymorphisms in shade avoidance

We reasoned that those non-synonymous polymorphisms in the *PHYB* coding region can contribute discretely to SAR. To assess this, we complemented the *phyB-9* null mutant in the Col-0 background with the 35S promoter transgenes expressing single or combined Pat-*PHYB* polymorphisms into the Col-*PHYB* cDNA fused to the Hemagglutinin tag (HA), as previously described in Sánchez-Lamas et al. (2016). As the control, we used the transgenic lines with Col-*PHYB* cDNA fused to the HA tag. We generated 12 transgenic lines, four of them individually expressing the previously mentioned Pat-*PHYB* polymorphisms (M1, M2, M3, and M4), and two additional lines expressing the combination of the two polymorphisms in the N-termini PSM (M1 + M2) and C-termini CTM (M3 + M4) to evaluate the functional relevance of both modules. At least two independent transgenic events were done for each mutation (M1, M2, M3, and M4) or combination (M1 + M2 and M3 + M4). We validated the quality of the generated material by measuring the mRNA levels of the transgenes (Supplementary Figure S5), and the protein levels in transgenic lines using a monoclonal anti-HA coupled to peroxidase (Roche 3F10; 2013819; Supplementary Figure S6).

**Figure 3:**
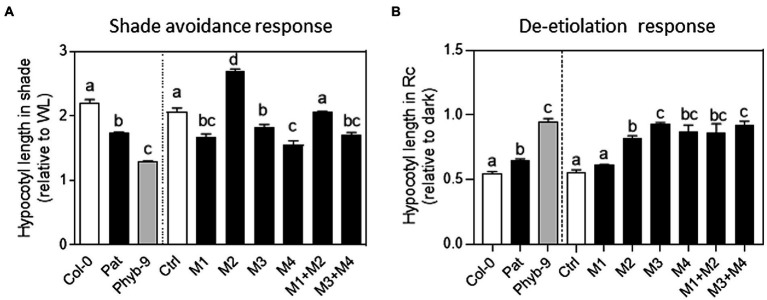
Functional variation of *PHYB* polymorphisms in shade avoidance response (SAR) and seedling de-etiolation response. **(A)** SAR index calculated as the ratio of hypocotyl length in simulated shade and WL. Each bar represents Mean ± SE (*n* = 4). Significant differences between means are shown with different letters by one-way ANOVA followed by Bonferroni post-test (*p* < 0.05). **(B)** Inhibition of hypocotyl length in red continuous light (relative to darkness) during seedling de-etiolation. Each bar represents Mean ± SE (*n* = 4). Significant differences between means are shown with different letters by one-way ANOVA followed by Bonferroni post-test (*p* < 0.05).

We evaluated the contribution of Pat-*PHYB* polymorphisms in the SAR. After de-etiolation, seedlings were exposed to WL or simulated shade for 5 days. We measured the hypocotyl length in WL, and simulated shade to estimate the SAR index as the ratio between simulated shade and WL. The SAR index for Col-0 was 2.3 and for *phyB-9* was 1.3 (Figure 3A). As expected, Pat seedlings showed a reduced SAR of 1.7 (Figure 3A). Transgenic seedlings with the full version of Col-*PHYB* in the *phyB-9* background rescued the Col-type phenotype (Figure 3A). Transgenic lines with the Pat polymorphisms on the N-termini domain (M1 = GGGR deletion) and the two polymorphisms on the C-termini domain (M3 = V980I and M4 = L1072V) showed a reduced SAR like Pat seedlings (Figure 3A). In opposition, transgenic seedlings with the M2 = I143L polymorphism showed a stronger SAR than the control (Figure 3A). The combination of Pat polymorphisms in the PSM rescued the phenotype of the control line suggesting that M1 and M2 operate in opposite manner (Figure 3A), and this is evident in WL condition (Supplementary Figure S7). We conclude that the I143L non-synonymous polymorphism promotes and the other Pat-*PHYB* polymorphisms reduce the SAR.

### Discrete functions of *PHYB* polymorphisms for seedling de-etiolation

We studied the role of *PHYB* polymorphisms in seedling de-etiolation regarding its known function in the inhibition of hypocotyl growth in R light (Casal et al., 1998). In continuous red light, the hypocotyl inhibition was around 50% in Col-0 and completely impaired in *phyB-9* seedlings (Figure 3B). Pat seedlings showed an intermediate light hyposensitive response being the hypocotyl inhibition around 65% under continuous red light. The transgenic seedlings with the Col-*PHYB* in *the phyB-9* background showed a similar response to Col-0 (Figure 3B). Seedlings with non-synonymous Pat-*PHYB* polymorphisms showed an impaired response like *phyB-9* seedlings except for the GGGR deletion polymorphism (i.e., M1, Figure 3B). In fact, continuous red light was most effective to inhibit hypocotyl length in M1 than those transgenic lines with other *PHYB* polymorphisms (Supplementary Figure S8). These results confirm that the three non-synonymous *PHYB*-Pat polymorphisms are responsible for the low red sensitivity response in seedling de-etiolation.

### Discrete functions of *PHYB* polymorphisms for seed germination

**Figure 4:**
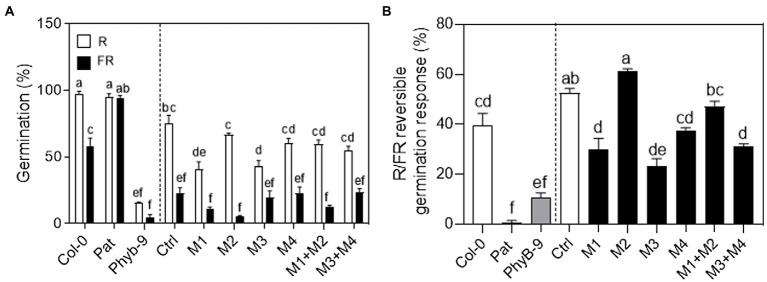
Functional variation of *PHYB* polymorphisms in seed germination. **(A)** Germination (%) with an R or FR saturated pulse and then incubated in darkness for 4 days at 25°C before germination was counted. The seeds were imbibed in water for 3 days at 7°C before the light pulse. **(B)** R/FR reversible response (%) calculated as the germination with a red pulse minus the germination with a far-red pulse. Mean ± SE (*n* = 6). Significant differences between means are shown with different letters by Two-way ANOVA **(A)** or One-way ANOVA **(B)** followed by Bonferroni post-test (*p* < 0.05).

The phyB is the principal photoreceptor mediating seed germination promoted by red light (Shinomura et al., 1994; Botto et al., 1995; Lee et al., 2012; Arana et al., 2014; Tognacca et al., 2019). We studied the contribution of the Pat-*PHYB* polymorphisms in the R/FR reversible response of seed germination (Figure 4). We evaluated the germination with a saturated far-red pulse (FRp) or red pulse (Rp) after incubation with chilling at 7°C for 3 days. Col-0 seeds showed an R/FR reversible response of 40%, while Pat seeds germinated independently of the light pulse suggesting that the light-dependent dormancy is completely lost (Figure 4). As expected, seeds of *phyb-9* null mutant did not germinate with an Rp or FRp (Figure 4A). The low seed germination induced by light is a common feature for all transgenic lines and this is evident when seeds were exposed to a FR pulse suggesting that they are more dormant than Col-0 and Pat seeds (Figure 4A; Supplementary Figure S9). Control transgenic seeds that expressed the complete version of the Col-*PHYB* showed 60% for the R/FR reversible response like Col-0 seeds (Figure 4B). Except for M2 and M1 + M2 transgenic lines, the rest of the mutations showed a reduced R/FR reversible response compared with the control (Figure 4B). M1 + M2 seeds rescued the germination phenotype of the WT control showing that both polymorphisms operate antagonistically as it was documented previously for the SAR (Figure 3A). We conclude that the GGGR deletion and I143L polymorphisms contribute oppositely to the R/FR reversible of seed germination, while V980I and L1072V polymorphisms on the C-termini are responsible for the reduced light sensitivity in seed germination mediated by the phyB.

### *PHYB* polymorphisms on the promoter are not responsible for light hyposensitivity

**Figure 5:**
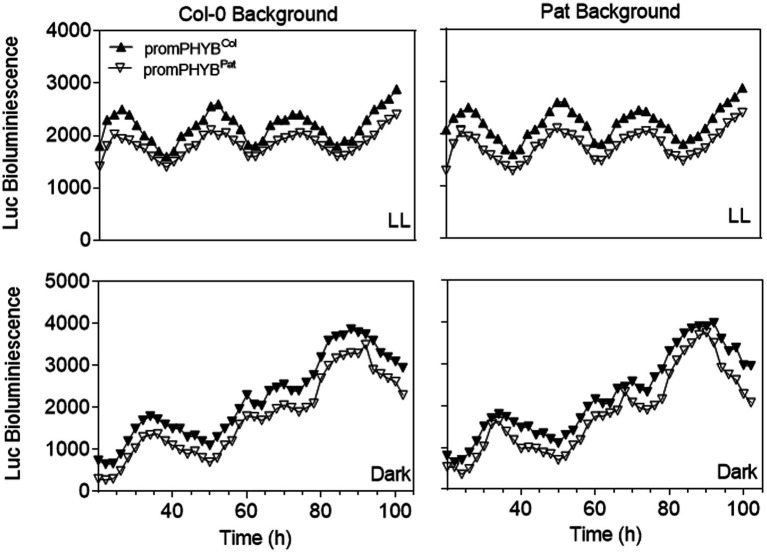
Functional analysis of *PHYB* polymorphisms on the promoter region by LUC analysis. Luc bioluminescence (units) for transgenic plants carrying the promotor of *PHYB*-Col or *PHYB*-Pat in the Col and Pat backgrounds. LL continuous light treatment. Each point represents the media of *n* ≥ 4.

We studied the function of the two indels Pat-*PHYB* polymorphisms on the promoter (i.e., the two-base insertion and the eight-base deletion at −318/−319 and −334/−341, respectively; Figure 2C). Two previous reports suggested that the phyB forms a regulatory loop within the circadian clock, besides setting the clock by transducing the light signal to the central oscillator. In fact, It has been demonstrated that PHYB::LUC reporter lines showed strong circadian oscillations under free running conditions under the control of the circadian clock (Bognár et al., 1999; Tóth et al., 2001). Regarding these previous results, we generated transgenic plants with the Pat and Col-0 promoters fused to the firefly Luciferase (LUC) in both genetic backgrounds to evaluate the function of the two polymorphisms of *PHYB* in the control of the circadian oscillations of the clock. We studied the circadian expression of the LUC reporter in continuous light and darkness for 4 days (Figure 5). Both promoter constructs behaved similarly in different genetic backgrounds. Together with previous reports In the expression of *PHYB* mRNA in Col-0 and Pat backgrounds (Kasulin et al., 2017), these results suggest that the polymorphisms on the promoter of *PHYB* between Col-0 and Pat are not responsible for the reduced light responses in the Pat accession.

## Discussion

Pat accession shows a reduced response to light (Kasulin et al., 2017; Matsusaka et al., 2021). The genetic architecture of the SAR was explored by QTL mapping in a new 162 RIL population between Col-0 and Pat (Figure 1). *PHYB* (At2g18790, 8,139–8,144 kb) is the gene candidate for *SAR1* QTL (LOD = 3.9, Var = 8.2%) because (i) *PHYB* gene falls into the 7,800–8,300 kb confidence interval of *SAR1* QTL, (ii) *PHYB*-Col-0 alleles at *SAR1* QTL contributes positively to the SAR, and (iii) the Pat phenotype is hyposensitive to light resembling the *phyB* mutant phenotype in WL (Figure 1; Table 1). *SAR1* QTL was mapped in the middle of chromosome II and colocalizes with other QTL for light traits mapped in different RIL populations. Kasulin et al. (2013) suggest that the *PHYB* gene is responsible for the FR-EOD shade response in Ler × Cvi, but not in Ler × Col and Bay × Sha RIL populations. These results reflect the relevance of the genetic background and the environmental context for the expression of the QTL. More recently, a study with a NAM population of Arabidopsis plants highlights the importance of an integrated view of the genotype–phenotype relationship accounting for genetics and environment together with phenotype relationships among shade avoidance traits throughout time (Ta et al., 2020).

*PHYB* was also mapped for seedling de-etiolation into the interval of a QTL mapped in WL and red continuous (Rc) light in the Ler × Cvi RIL population (Borevitz et al., 2002). Furthermore, a contrasting response for seedling de-etiolation in Rc light was documented in transgenic lines with Ler-*PHYB* and Cvi-*PHYB* alleles (Filiault et al., 2008). Interestingly, *SAR1* QTL mapped close to *Rc2* QTL for seedling de-etiolation in Rc light in the novel Col-0 × Pat RIL population used in this study (Matsusaka et al., 2021), but recombination points in RIL lines allowed the dissection of two independent QTLs (Supplementary Figure S3). Previously, *PHYB* was also mapped close, but outside, of the *RED2* QTL interval for seedling de-etiolation in Rc light in the Col-gl1 × Kas1 RIL population (Wolyn et al., 2004). Overall, these results suggest that the SAR and the seedling de-etiolation are developmental processes mediated by the phyB and by other genetic regulators which map remarkably close to *PHYB* depending on the genetic backgrounds.

PHYB apoprotein variants are responsible for phenotypic differences between Pat and Col-0 accessions. Here, we evaluated individual and combined contributions of *PHYB* polymorphisms that mapped into the same functional module (PSM and CTM). Globally, our results suggest that (i) Pat-*PHYB* polymorphisms have discrete functions in seed germination, seedling de-etiolation, and shade avoidance response; (ii) *PHYB-Pat* polymorphisms on both PSM and CTM contribute to the hyposensitive light response; and (iii) the Pat polymorphisms have positive, neutral or negative effects depending on the phyB-mediated response. Pat polymorphisms on the CTM (i.e., V980I and L1072V) always reduce the light response independently of the physiological response (Figures 3, 4). However, Pat polymorphisms on the PSM can show opposite effects according with the physiological response. For example, the GGGR deletion at the N-termini extension in Pat-*PHYB* reduces the light response in germination and shade, while the I143L promotes the shade avoidance response (Figures 3A, 4). The present study corroborates the results of Nicholson et al. (2011) indicating that a GGGR deletion at 9–12 position in the N-termini extension of Col-0 reduces the activity of the phyB in WL and R light. Interestingly, it has been demonstrated the pivotal role of the 1–90 amino-acid N-termini extension of serine-rich region, proximal to the PAS-GAF interface, for the phyB Pfr stability impeding the binding to PIF3 (Rockwell and Lagarias, 2006; Oka et al., 2008).

The function of the PAS domain in the photosensory module where locates the I143L polymorphism is currently unclear. It has been suggested that the PAS and GAF tight connection *via* the knots provides a backstop to direct motion from the GAF domain towards the PHY domain (Burgie et al., 2014). Interestingly, the I143L polymorphism has contrasting effects in the control of hypocotyl growth in seedling de-etiolation and SAR (Figure 3) being this polymorphism crucial for the phytochrome interactions with downstream signaling proteins instead of altered photobiological phytochrome properties.

The CTM is involved in dimerization, nuclear import, and localization into subnuclear photobodies structures, and also engages in light-regulated interactions and posttranscriptional modifications with SUMOylation limiting the ability of active phyB to interact with downstream signaling targets (Legris et al., 2019). The two Pat-*PHYB* polymorphisms belonging to the CTM module located in the plant-specific HKRD domain contribute to reducing the response to light. Pat polymorphisms at V980I and L1072V affect seedling de-etiolation and SAR in an epistatic manner suggesting that the same protein interactions can be altered (Figure 3).

This work demonstrates that the *PHYB*-Pat polymorphisms generate functional variants that account for the light sensitivity responses correlated with the phenotypes observed in the colonization areas of Pat in Patagonia (Kasulin et al., 2017), and suggests a versatile function of the individual *PHYB* polymorphisms for the adjustment of plant development to variable light conditions. We conclude that the study of polymorphisms of relevant genes in plant signaling and development might contribute to understanding the natural genetic basis of plant’s adaptation to their environment.

## Data availability statement

The datasets presented in this study can be found in online repositories. The names of the repository/repositories and accession number(s) can be found at: NCBI GenBank – NC 003071, NM_127435.3.

## Author contributions

JB conceived the project and designed the experiments. MR-D and DM performed the experiments with the assistance of JC and MS-L for gene constructions. DM and DS performed QTL mapping. PC contributed with reagents and materials. MR-D, PC, and JB analyzed the data. JB wrote the manuscript with the contribution of all the authors. All authors contributed to the article and approved the submitted version.

## Funding

This wok was supported by grants from the University of Buenos Aires to JB (UBACYT 20020170100265BA and SPU VT38-UBA9537) and the Agencia Nacional de Promoción Científica y Tecnológica of Argentina (PICT2017-0583 and PICT2019-2807).

## Conflict of interest

The authors declare that the research was conducted in the absence of any commercial or financial relationships that could be construed as a potential conflict of interest.

## Publisher’s note

All claims expressed in this article are solely those of the authors and do not necessarily represent those of their affiliated organizations, or those of the publisher, the editors and the reviewers. Any product that may be evaluated in this article, or claim that may be made by its manufacturer, is not guaranteed or endorsed by the publisher.

## Supplementary material

SUPPLEMENTARY FIGURE S1 Light spectrums used in the work. **(A)** white light, **(B)** shade light, **(C)** red light and **(D)** far-red light.

SUPPLEMENTARY FIGURE S2 Frequency distribution for WL, simulated shade, and SAR index was calculated as the ratio of hypocotyl length in the simulated shade and WL in the Col-0 and Pat RIL population. The average response in each light condition of parental lines is indicated with arrows (red= Col-0 and blue= Pat).

SUPPLEMENTARY FIGURE S3 Confirmation of *SHADE1* QTL in the simulated shade by two independent HIFs. The markers used for the selection of HIF20 and HIF71 include the interval between 25.089 kb and 26.796 kb into the chromosome V. For more information about the construction of both HIFs see Matsusaka et al., 2021.

SUPPLEMENTARY FIGURE S4 QTL mapping for Rc/dark (data from Matsusaka et al., 2021) and SAR index (data from this study) in the or Col-0 × Pat RIL population. LOD values for each trait corresponding to chromosome II. Note that the confidence interval of Rc/Dark QTL falls outside of the *SAR1* QTL where maps *PHYB* gene. LOD score is shown for the absolute distance of chromosome II (Mb).

SUPPLEMENTARY FIGURE S5 qPCR *PHYB* gene expression in Col-0, Pat and transgenics plants. The *PHYB* expression was estimated in at least two independent lines generated for each mutation. Ctrl = *PHYB*-Col-0 into *phyb-9* null background, M1= GGGR deletion, M2= I143L, M3= V980I, and M4= L1072V, M5= M1+M2 and M6= M3+M4 correspond to transgenic lines with single or combined *PHYB*-Pat polymorphisms.

SUPPLEMENTARY FIGURE S6 Dot-Blot for the quantification of PHYB protein of independent transgenic lines for each polymorphism. (A) Western blot for each representative polymorphism of PHYB. The blot shows the PHYB intensity band for each genotype, ctrl+: PHYB-Col-0 in phyB-9, ctrol-: the empty vector in phyB-9. (B) Calibration curve between signal intensity and HA concentration. The Ag sensor was loaded at concentrations ranging from 160 ng/ml to 20 μg/ml. Samples were loaded at different dilutions as indicated on a representative blot for duplicate. The adjust of the regression of the averaged signal with the calibration sensor was estimated *R*^2^ = 0.9508. (C) The HA concentration for PHYB quantification in each transgenic line is calculated with the parameters obtained in (B). (D) Dot blot of independent transgenic lines for each polymorphism at different dilutions.

SUPPLEMENTARY FIGURE S7 Functional variation of *PHYB* polymorphisms in shade avoidance response (SAR). Hypocotyl length in white light and shade light (A) or the SAR index (B) calculated as the ratio of hypocotyl length in simulated shade and WL for each PHYB polymorphism in independent transgenic lines. Mean ± SE (n=2).

SUPPLEMENTARY FIGURE S8 Functional variation of *PHYB* polymorphisms in seedling de-etiolation response. Hypocotyl length in darkness and red continuous light (A) or the de-etiolation inhibition response (B) calculated as the ratio of hypocotyl length in red continuous and darkness for each PHYB polymorphism in independent transgenic lines. Mean ± SE (n=2).

SUPPLEMENTARY FIGURE S9 Functional variation of *PHYB* polymorphisms in seed germination induced by a red or far-red pulse for each PHYB polymorphism in independent transgenic lines. Mean ± SE (*n* = 3).

SUPPLEMENTARY TABLE S7 SNPs into the *PHYB* gene detected in the five samples of RNAseq of Patagonia (for more references see Kasulin et al., 2017). The table shows the SNPs in the promoter (−2,000b), 5′UTR, exons, and introns of the *PHYB*. The three non-synonymous polymorphisms detected in the cDNA of the *PHYB* correspond to M2 (I143L), M3 (V980I), and M4 (L1072V).
