# Supplementary material for: Functional analysis of PHYB polymorphisms in Arabidopsis thaliana collected in Patagonia
Source: Front Plant Sci. 2022 Sep 7;13:952214. doi: 10.3389/fpls.2022.952214 (PMC9490419; doi:10.3389/fpls.2022.952214)
Supplement: SUPPLEMENTARY FIGURE S1 — Light spectrums used in the work. (A) white light, (B) shade light, (C) red light and (D) far-red light. [file Data_Sheet_1.PDF]

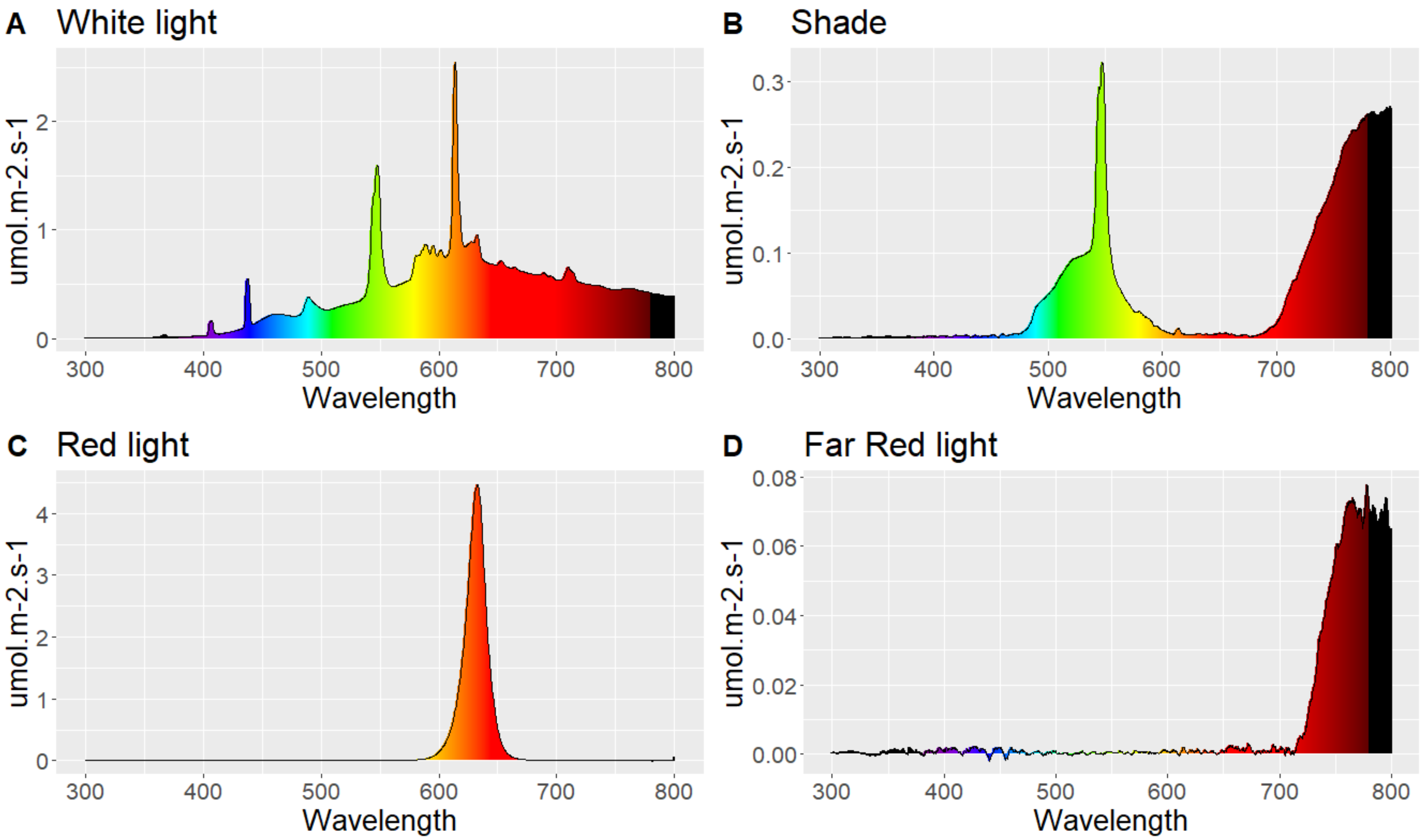
A White light
B Shade
C Red light
D Far Red light
umol.m-2.s-1
Wavelength
0
1
2
3
4
0.0
0.1
0.2
0.3
0.00
0.02
0.04
0.06
0.08
300
400
500
600
700
800

Figure S1.

A. WL and shade

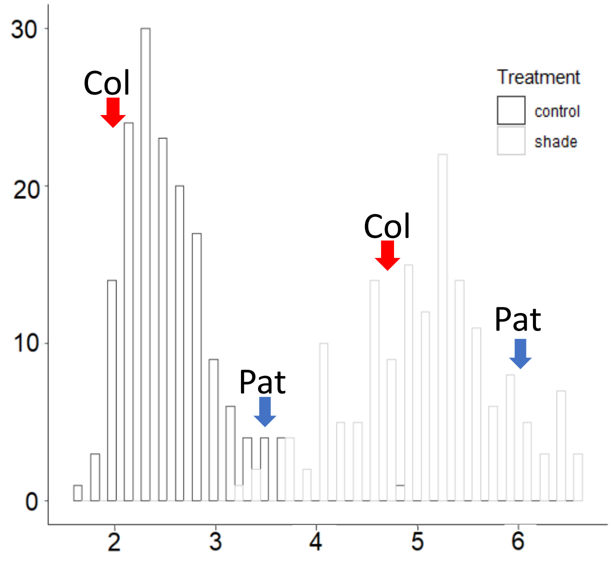

B. Shade avoidance response (SAR index= Shade/WL)

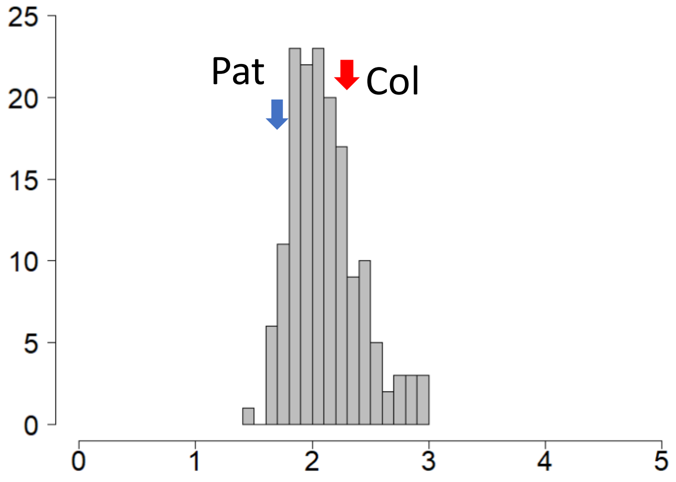

Figure S2.

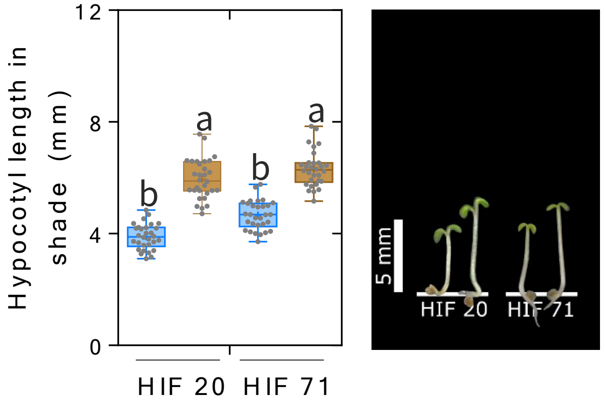
Hypocotyl length in shade (mm)
12
8
4
0
b
a
b
a
HIF 20
HIF 71
5 mm
HIF 20
HIF 71

Figure S3.

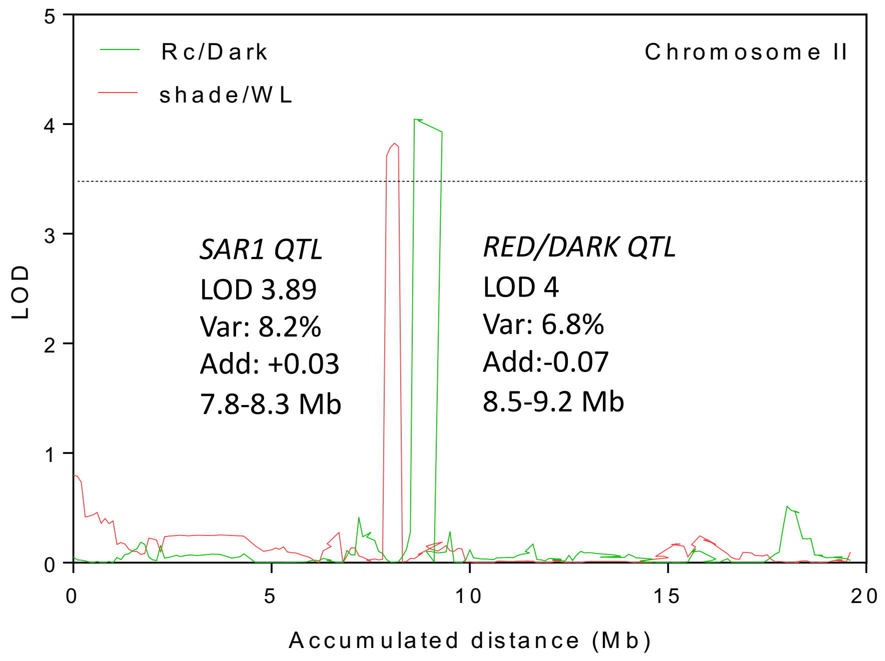
Rc/Dark
shade/WL
Chromosome II
SAR1 QTL
LOD 3.89
Var: 8.2%
Add: +0.03
7.8-8.3 Mb
RED/DARK QTL
LOD 4
Var: 6.8%
Add:-0.07
8.5-9.2 Mb
LOD
0
1
2
3
4
5
0
5
10
15
20
Accumulated distance (Mb)

Figure S4.

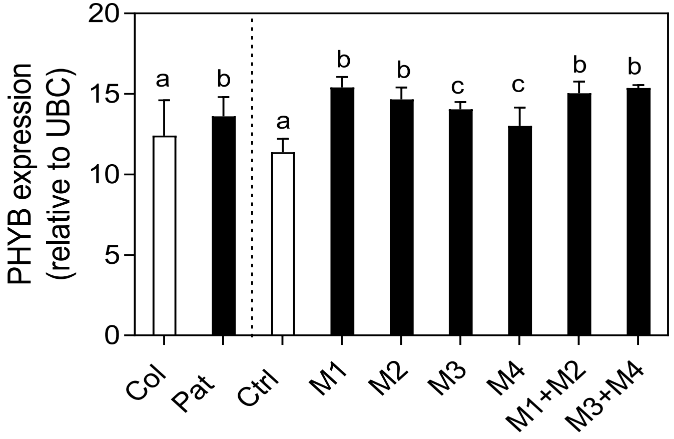

PHYB expression
(relative to UBC)
20
15
10
5
0
a
b
a
b
b
c
c
b
b
Col
Pat
Ctrl
M1
M2
M3
M4
M1+M2
M3+M4

Figure S5.

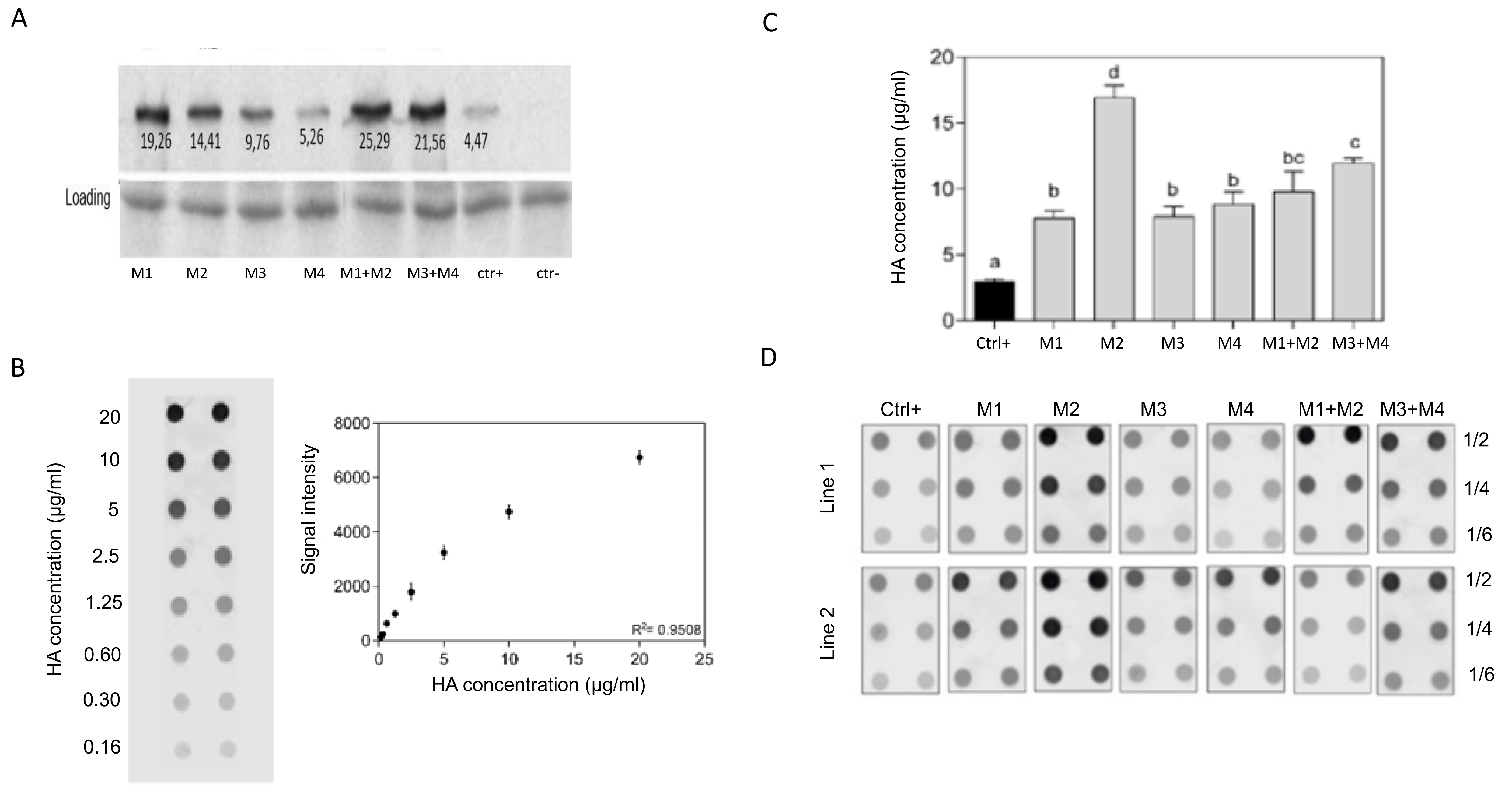
A
19,26
14,41
9,76
5,26
25,29
21,56
4,47
Loading
M1
M2
M3
M4
M1+M2
M3+M4
ctr+
ctr-
B
HA concentration (µg/ml)
20
10
5
2.5
1.25
0.60
0.30
0.16
Signal intensity
8000
6000
4000
2000
0
0
5
10
15
20
25
HA concentration (µg/ml)
R²= 0.9508
C
HA concentration (µg/ml)
20
15
10
5
0
a
b
d
b
b
bc
c
Ctrl+
M1
M2
M3
M4
M1+M2
M3+M4
D
Ctrl+
M1
M2
M3
M4
M1+M2
M3+M4
Line 1
Line 2
1/2
1/4
1/6
1/2
1/4
1/6

Figure S6.

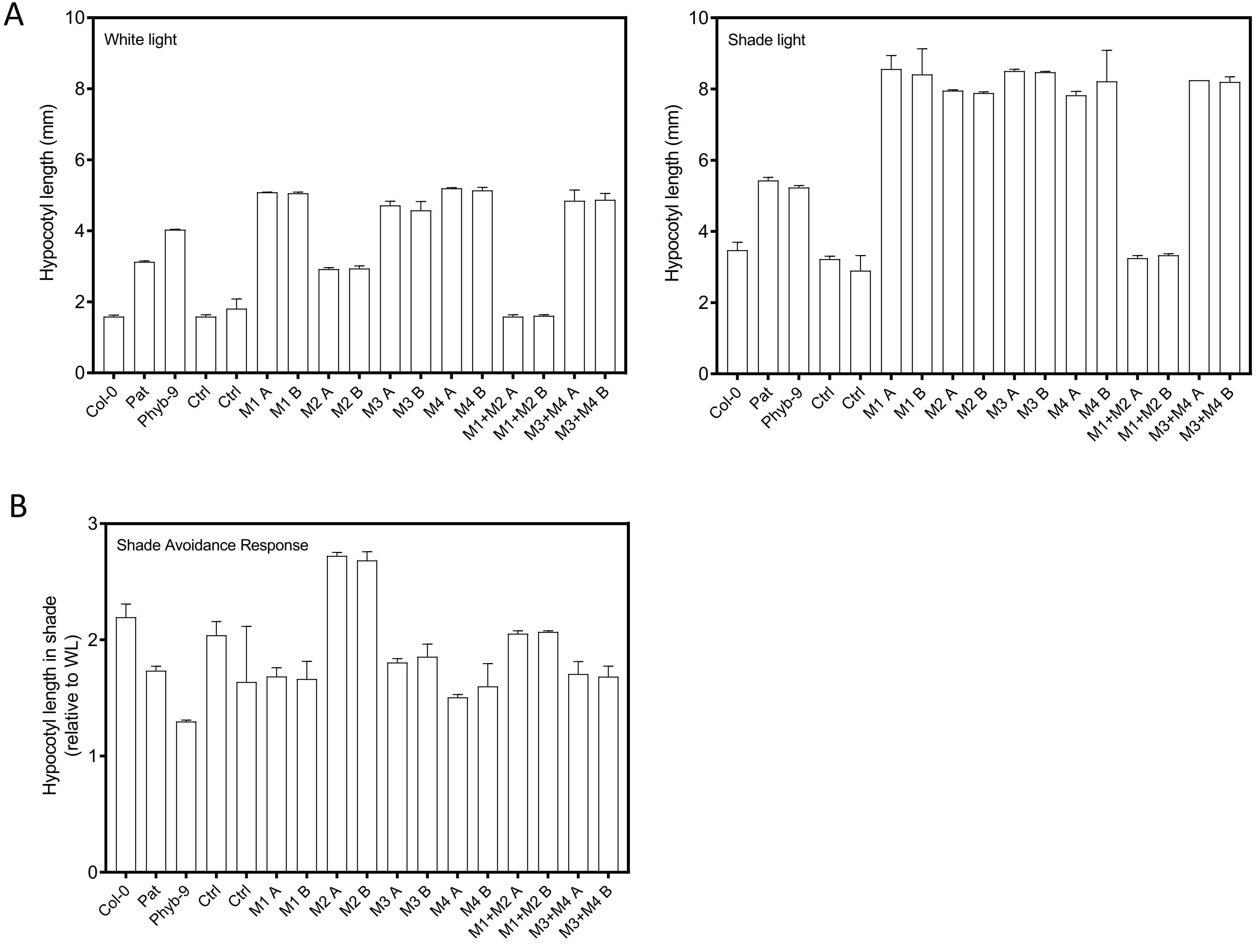
A
White light
Hypocotyl length (mm)
0
2
4
6
8
10
Col-0
Pat
Phyb-9
Ctrl
Ctrl
M1 A
M1 B
M2 A
M2 B
M3 A
M3 B
M4 A
M4 B
M1+M2 A
M1+M2 B
M3+M4 A
M3+M4 B
Shade light
Hypocotyl length (mm)
0
2
4
6
8
10
Col-0
Pat
Phyb-9
Ctrl
Ctrl
M1 A
M1 B
M2 A
M2 B
M3 A
M3 B
M4 A
M4 B
M1+M2 A
M1+M2 B
M3+M4 A
M3+M4 B
B
Shade Avoidance Response
Hypocotyl length in shade
(relative to WL)
0
1
2
3
Col-0
Pat
Phyb-9
Ctrl
Ctrl
M1 A
M1 B
M2 A
M2 B
M3 A
M3 B
M4 A
M4 B
M1+M2 A
M1+M2 B
M3+M4 A
M3+M4 B

Figure S7.

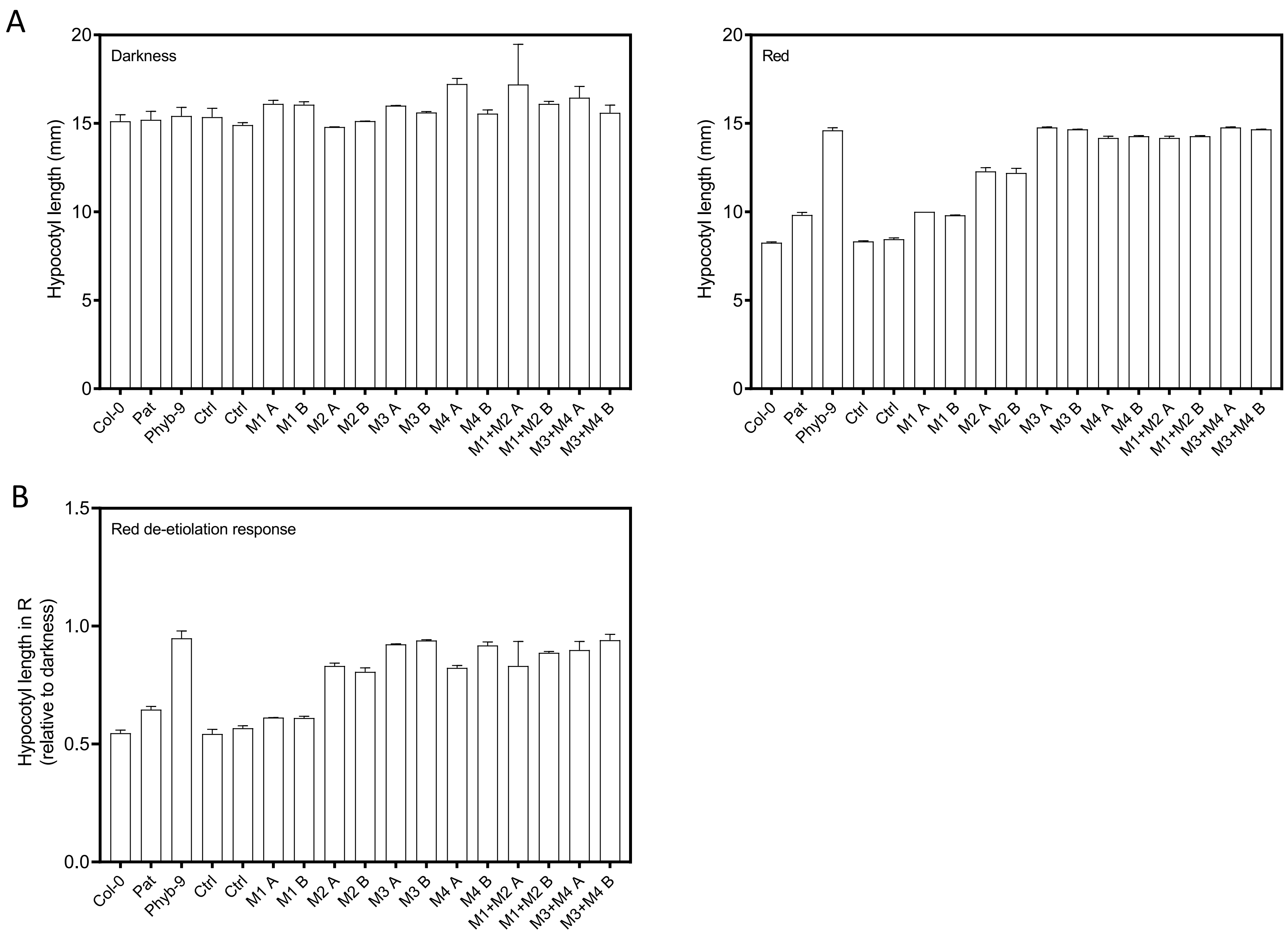
A
Darkness
Hypocotyl length (mm)
Red
Hypocotyl length (mm)
B
Red de-etiolation response
Hypocotyl length in R (relative to darkness)
Col-0
Pat
Phyb-9
Ctrl
Ctrl
M1 A
M1 B
M2 A
M2 B
M3 A
M3 B
M4 A
M4 B
M1+M2 A
M1+M2 B
M3+M4 A
M3+M4 B

Figure S8.

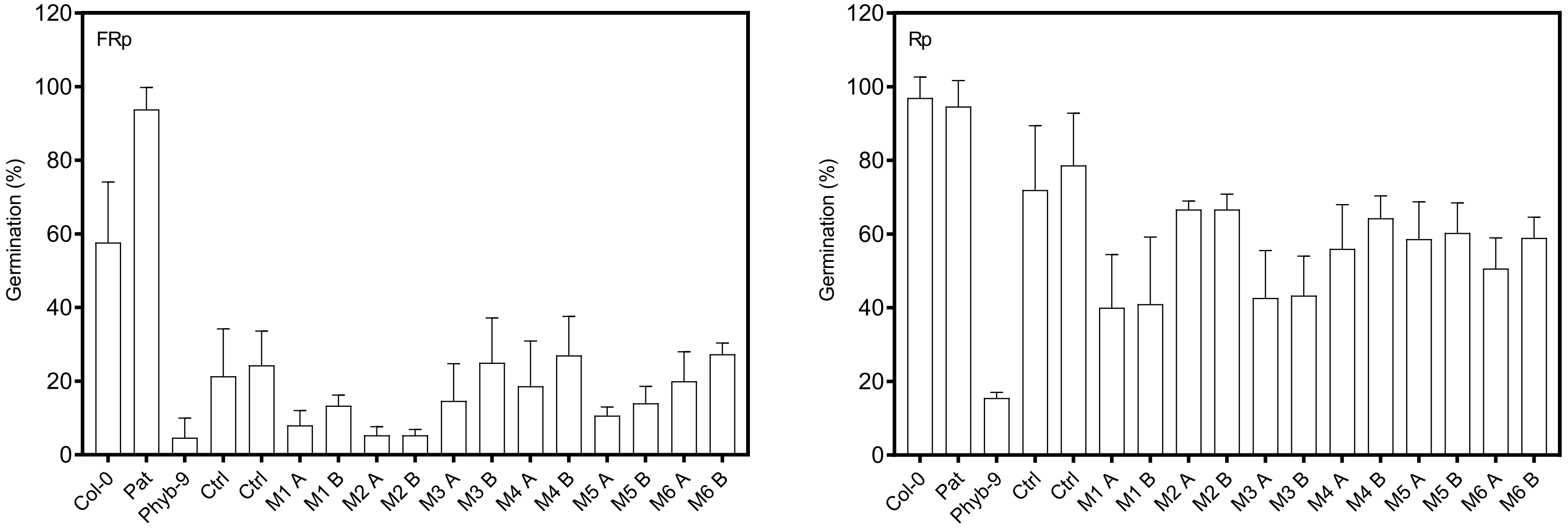

FRp
Rp
Germination (%)
0
20
40
60
80
100
120
Col-0
Pat
Phyb-9
Ctrl
Ctrl
M1 A
M1 B
M2 A
M2 B
M3 A
M3 B
M4 A
M4 B
M5 A
M5 B
M6 A
M6 B

Figure S9.
