# Supplementary material for: Functional analysis of PHYB polymorphisms in Arabidopsis thaliana collected in Patagonia
Source: Front Plant Sci. 2022 Sep 7;13:952214. doi: 10.3389/fpls.2022.952214 (PMC9490419; doi:10.3389/fpls.2022.952214)
Supplement: SUPPLEMENTARY TABLE S7 — SNPs into the PHYB gene detected in the five samples of RNAseq of Patagonia (for more references see Kasulin et al., 2017). The table shows the SNPs in the promoter (−2,000b), 5′UTR, exons, and introns of the PHYB. The three non-synonymous polymorphisms detected in the cDNA of the PHYB correspond to M2 (I143L), M3 (V980I), and M4 (L1072V). [file Data_Sheet_2.zip › Table S1.docx]

Table S1: List of accessions and mutant used in this study with the reference code from the ABRC.

| **Accession**  Col-0  Pat  phyb-9 | **Source**  ABRC  ABRC  ABRC | **Ref., no.**  CS28167  CS79057  CS6217 |
| --- | --- | --- |
| Agu-1 | ABRC | CS76409 |
| Aru-0 | ABRC | CS76674 |
| Bes-5 | ABRC | CS76702 |
| Bla-1 | ABRC | CS28079 |
| Boa-0 | ABRC | CS76714 |
| Di-g | ABRC | CS910 |
| Dr-0 | ABRC | CS28211 |
| Ei-2 | ABRC | CS6689 |
| Hey-1 | ABRC | CS78245 |
| Kävl-1 | ABRC | CS76964 |
| Kelst-4 | ABRC | CS78546 |
| Ler-2 | ABRC | CS8581 |
| Mt-0 | ABRC | CS6799 |
| Nd-1 | ABRC | CS1636 |
| No-0 | ABRC | CS3081 |
| RLD1 | ABRC | CS913 |
| Sp-0 | ABRC | CS28743 |
| Su-0 | ABRC | CS6866 |
| Uk-4 | ABRC | CS6882 |
| War-0 | ABRC | CS8144 |
| Wei-0 | ABRC | CS6182 |
