# Supplementary material for: Functional analysis of PHYB polymorphisms in Arabidopsis thaliana collected in Patagonia
Source: Front Plant Sci. 2022 Sep 7;13:952214. doi: 10.3389/fpls.2022.952214 (PMC9490419; doi:10.3389/fpls.2022.952214)
Supplement: SUPPLEMENTARY TABLE S7 — SNPs into the PHYB gene detected in the five samples of RNAseq of Patagonia (for more references see Kasulin et al., 2017). The table shows the SNPs in the promoter (−2,000b), 5′UTR, exons, and introns of the PHYB. The three non-synonymous polymorphisms detected in the cDNA of the PHYB correspond to M2 (I143L), M3 (V980I), and M4 (L1072V). [file Data_Sheet_2.zip › Table S3.docx]

Table S3: Statistics summary for hypocotyl length in WL, shade and shade avoidance response (SAR) calculated as the shade/WL index in the Col-0 x Pat RIL population.

|  | WL (mm) | Shade (mm) | Shade response  (Shade/WL) |
| --- | --- | --- | --- |
| Pat | 3.45 ± 0.11 | 5.99 ± 0.09 | 1.73 ± 0.02 |
| Col-0 | 2.19 ± 0.03 | 4.63 ± 0.14 | 2.11 ± 0.06 |
| RIL mean | 2.49 ± 0.04 | 5.08 ± 0.08 | 2.11 ± 0.03 |
| RIL max–min | 4.3 - 1.75 | 6.63 - 3.41 | 2.93 - 1.47 |
| CMG | 0.49 | 1.48 | 0.24 |
| CME | 0.14 | 0.25 | 0.12 |
| VG | 0.17 | 0.41 | 0.04 |
| VP | 0.16 | 0.49 | 0.08 |
| H_2_ | 0.71 | 0.83 | 0.50 |

CMG is the variance component for each trait among RIL lines.

CME is the residual (error) variance component for each trait among RIL lines.

VG is the genotypic variance for each trait among RIL lines.

VP is the phenotypic variance for each trait among RIL lines.

H_2_ is defined as heritability that is the measure of VG/VP.
