# Supplementary material for: Functional analysis of PHYB polymorphisms in Arabidopsis thaliana collected in Patagonia
Source: Front Plant Sci. 2022 Sep 7;13:952214. doi: 10.3389/fpls.2022.952214 (PMC9490419; doi:10.3389/fpls.2022.952214)
Supplement: SUPPLEMENTARY TABLE S7 — SNPs into the PHYB gene detected in the five samples of RNAseq of Patagonia (for more references see Kasulin et al., 2017). The table shows the SNPs in the promoter (−2,000b), 5′UTR, exons, and introns of the PHYB. The three non-synonymous polymorphisms detected in the cDNA of the PHYB correspond to M2 (I143L), M3 (V980I), and M4 (L1072V). [file Data_Sheet_2.zip › Table S4.docx]

Table S4: Annotated genes within each QTL interval according to Araport11.

| **QTL** | **# genes per QTL** |
| --- | --- |
| *WL1* | 218 |
| *WL2* | 399 |
| *SHADE1* | 143 |
| *SAR1* | 132 |
| total | 892 |
