# Supplementary material for: Functional analysis of PHYB polymorphisms in Arabidopsis thaliana collected in Patagonia
Source: Front Plant Sci. 2022 Sep 7;13:952214. doi: 10.3389/fpls.2022.952214 (PMC9490419; doi:10.3389/fpls.2022.952214)
Supplement: SUPPLEMENTARY TABLE S7 — SNPs into the PHYB gene detected in the five samples of RNAseq of Patagonia (for more references see Kasulin et al., 2017). The table shows the SNPs in the promoter (−2,000b), 5′UTR, exons, and introns of the PHYB. The three non-synonymous polymorphisms detected in the cDNA of the PHYB correspond to M2 (I143L), M3 (V980I), and M4 (L1072V). [file Data_Sheet_2.zip › Table S6.docx]

| **QTL** | **Gene** | **Name** | **Missense variant** | **Splice acceptor variant** | **Splice donor variant** | **Start lost** | **Stop gained** | **Stop lost** |
| --- | --- | --- | --- | --- | --- | --- | --- | --- |
| *SAR1* | AT2G18010 | AT2G18010 | 1 | 0 | 0 | 0 | 0 | 0 |
|  | AT2G18790 | PHYB | 3 | 0 | 0 | 0 | 0 | 0 |
|  | AT2G18915 | ADO2 | 3 | 0 | 0 | 0 | 0 | 0 |
| *SHADE1* | AT5G64330 | RPT3 | 1 | 0 | 0 | 0 | 0 | 0 |

Table S6: Light-responsive candidate genes mapped into *SAR1* and *SHADE1* QTLs. No candidate light-responsive genes were documented into the genetic interval of *WL1* and *WL2* QTL
